# Functional Genomic Analysis of Amphetamine Sensitivity in *Drosophila*

**DOI:** 10.3389/fpsyt.2022.831597

**Published:** 2022-02-18

**Authors:** Caline S. Karam, Brenna L. Williams, Irina Morozova, Qiaoping Yuan, Rony Panarsky, Yuchao Zhang, Colin A. Hodgkinson, David Goldman, Sergey Kalachikov, Jonathan A. Javitch

**Affiliations:** ^1^Department of Psychiatry, Columbia University Vagelos College of Physicians and Surgeons, New York, NY, United States; ^2^Division of Molecular Therapeutics, New York State Psychiatric Institute, New York, NY, United States; ^3^Center for Genome Technology and Biomolecular Engineering, Columbia University, New York, NY, United States; ^4^Department of Chemical Engineering, Columbia University, New York, NY, United States; ^5^Laboratory of Neurogenetics, National Institute on Alcohol Abuse and Alcoholism, Bethesda, MD, United States; ^6^Department of Molecular Pharmacology and Therapeutics, Columbia University Vagelos College of Physicians and Surgeons, New York, NY, United States

**Keywords:** *Drosophila*, dopamine transporter, DGRP, transcriptomic (RNA-Seq), amphetamine, psychostimulants, mammalian target of rapamycin (mTOR), S6K (70-kDa ribosomal protein S6 kinase)

## Abstract

Abuse of psychostimulants, including amphetamines (AMPHs), is a major public health problem with profound psychiatric, medical, and psychosocial complications. The actions of these drugs at the dopamine transporter (DAT) play a critical role in their therapeutic efficacy as well as their liability for abuse and dependence. To date, however, the mechanisms that mediate these actions are not well-understood, and therapeutic interventions for AMPH abuse have been limited. Drug exposure can induce broad changes in gene expression that can contribute to neuroplasticity and effect long-lasting changes in neuronal function. Identifying genes and gene pathways perturbed by drug exposure is essential to our understanding of the molecular basis of drug addiction. In this study, we used *Drosophila* as a model to examine AMPH-induced transcriptional changes that are DAT-dependent, as those would be the most relevant to the stimulatory effects of the drug. Using this approach, we found genes involved in the control of mRNA translation to be significantly upregulated in response to AMPH in a DAT-dependent manner. To further prioritize genes for validation, we explored functional convergence between these genes and genes we identified in a genome-wide association study of AMPH sensitivity using the *Drosophila* Genetic Reference Panel. We validated a number of these genes by showing that they act specifically in dopamine neurons to mediate the behavioral effects of AMPH. Taken together, our data establish *Drosophila* as a powerful model that enables the integration of behavioral, genomic and transcriptomic data, followed by rapid gene validation, to investigate the molecular underpinnings of psychostimulant action.

## Introduction

The use of prescribed and illicit amphetamines (AMPHs) has been growing steadily, with an estimated 50 million worldwide users in 2017 (1). These drugs are widely abused, often leading to aggression, psychosis, cardiovascular damage, and a host of other medical and psychosocial complications (2, 3). AMPHs act as substrates for the dopamine transporter (DAT), which mediates the inactivation of released dopamine through reuptake. The actions of AMPHs lead to a dramatic increase of extracellular dopamine *via* non-exocytic efflux of dopamine through DAT-mediated reverse transport (4–7). This dopamine increase is believed to play a major role in the psychostimulatory and rewarding properties of AMPHs (4, 5, 8). To date, however, the mechanisms that mediate the transition from drug use to abuse are not fully understood, and therapeutic interventions for AMPH abuse have been limited and largely ineffective.

Neuroadaptations in response to drugs of abuse have been extensively reported (9–13). Specifically, studies have shown that drug exposure can induce broad changes in gene expression, which can contribute to neuroplasticity and effect long-lasting changes in neuronal function, ultimately leading to the development of drug-seeking behavior (11, 14). Identifying genes and gene pathways perturbed by drug exposure is essential to our understanding of the molecular basis of drug addiction and can help identify novel therapeutic targets and guide the development of novel treatment and prevention measures for substance use disorders.

Studies in humans encounter multiple challenges, including the difficulty of quantifying behavioral phenotypes presented by complex brain disorders, such as addiction, and gene x environment interactions, which can further confound results (15–17). To overcome these challenges, animal models have emerged as critical tools for investigating in a systematic manner the molecular and cellular mechanisms underlying the actions of drugs of abuse (18). Acute stimulation of locomotor behaviors is one of the most widely studied effects of psychostimulants. It has been suggested that acute drug effects in animals may model the initial sensitivity experienced by humans during early drug use. This initial sensitivity varies significantly among individuals (19) and has been associated with continued drug use (20–23). Similarly, in mice, differences in the sensitivity to the locomotor effects of methamphetamines are heritable (24) and, importantly, predict later self-administration (25, 26).

Using a *Drosophila* behavioral assay, we previously showed that flies respond to AMPH by increasing their locomotor activity (27, 28) and decreasing their sleep (27) in a dopamine-dependent manner. Flies that carry a loss-of-function mutation in the gene encoding the *Drosophila* DAT homolog (*dDAT*^*fmn*^, henceforth referred to as *DAT* mutants) display no detectable DAT in the brain and exhibit heightened activity levels at baseline, consistent with increased levels of extracellular dopamine caused by the impairment of reuptake (27, 29). Critically, our data showed that *DAT* mutant flies failed to increase their activity in response to AMPH (27), consistent with DAT being the principal molecular target for AMPH (4, 5). Taken together, these data demonstrate that we have developed a robust tool to associate molecular perturbations with the actions of AMPH *in vivo*. In this study, we used this model to examine the transcriptional changes induced by AMPH treatment. Since DAT is essential for the locomotor effects of AMPH (27), we focused our analysis on gene expression changes that were DAT-dependent by comparing the transcriptomes of *DAT* mutant flies to those of control flies, to identify genes that were associated specifically with the actions of AMPH at DAT, as those would be the most relevant to the stimulatory effects of the drug. To further prioritize genes for validation, we explored functional convergence between these genes and genes we identified in a genome-wide association study of AMPH sensitivity using the *Drosophila* Genetic Reference Panel (DGRP) (30). Using this approach, we identified several genes that play a role in modulating mRNA translation and processing. Taking advantage of the tools available in flies for targeted gene manipulation (31, 32), we validated a number of these genes by showing that they act specifically in dopamine neurons to mediate the behavioral effects of AMPH. Taken together, our data establish *Drosophila* as a powerful model that enables the integration of behavioral data, with transcriptomic data and GWAS, followed by rapid gene validation, to investigate the molecular underpinnings of psychostimulant action.

## Results

### Transcriptional Response to AMPH in *Drosophila*

**Figure 1:**
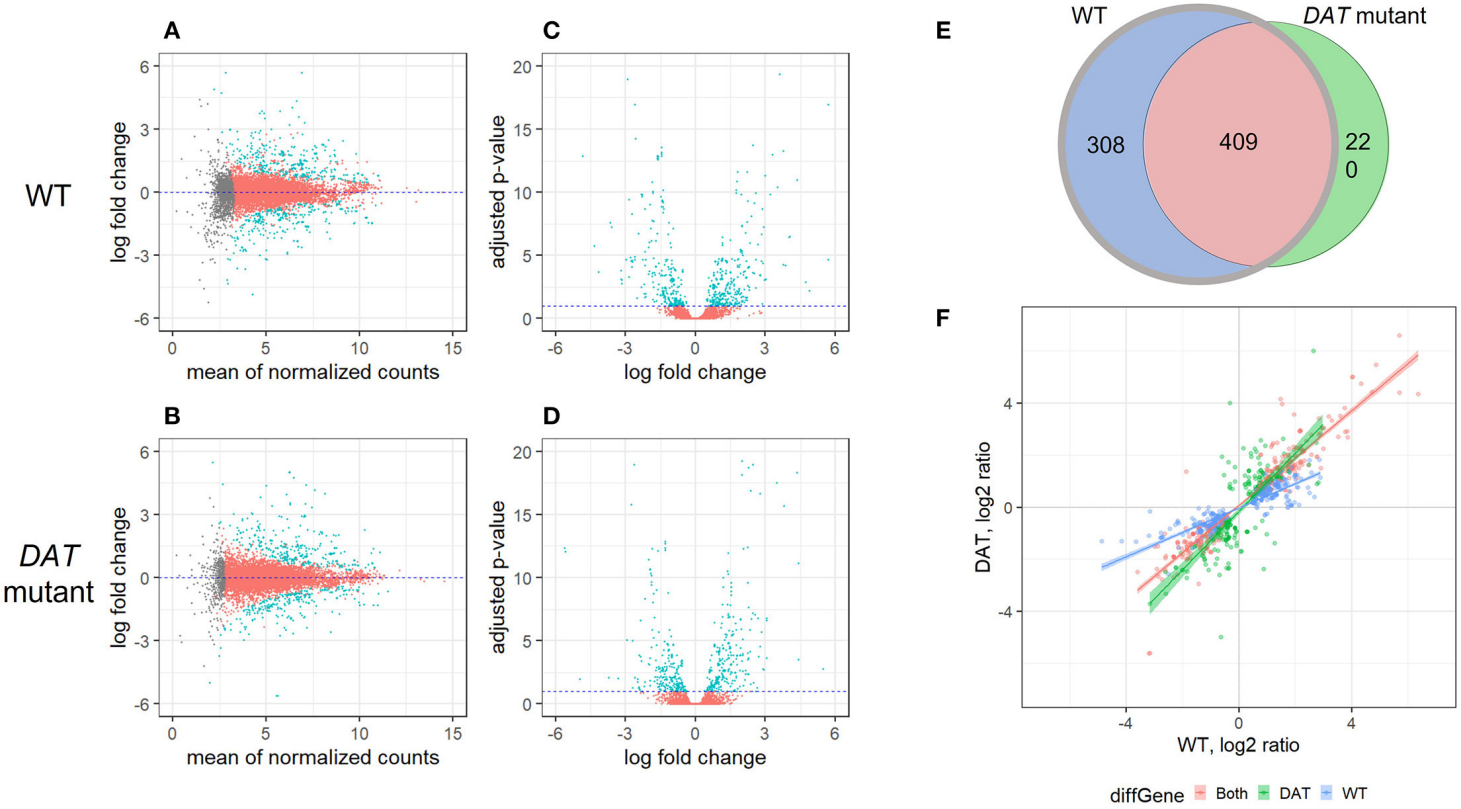
Differential gene expression in response to AMPH in *DAT* mutant and isogenic control. **(A–D)** are gene expression scatterplots for the two strains of flies: WT (*w*^1118^ isogenic strain) and *DAT* mutant. **(A,B)** are MA plots and **(C,D)** are volcano plots. In **(A–C)**, differentially expressed transcripts (DE transcripts, those that change their expression in treatment vs. vehicle) are shown in teal; transcripts with changes below the statistical cut-off are in red, while transcripts for which the differential expression status could not be resolved are shown in gray. **(E)** Overlap between the DE transcript sets between WT (gray circle) and *DAT* mutant (black circle). At FDR < 0.1, there were 717 DE transcripts in the WT strain and 629 DE transcripts in the *DAT* mutant flies. The two lists of DE transcripts had 308 genes that were DE only in WT (blue), 220 transcripts that were DE only in DAT (green) and 409 transcripts in common (red). **(F)** Comparison of the expression level changes between the WT and *DAT* mutant strains. The 409 DE transcripts shared between the two strains are shown in red; the 220 *DAT* mutant-specific DE transcripts are shown in green, and the 308 WT-specific DE transcripts are shown in blue.

To identify DAT-dependent changes in AMPH-induced gene expression, we performed RNA-seq on *Drosophila* head extracts from isogenic *w*^1118^ flies (WT) and flies carrying a loss-of-function mutation in the *Drosophila DAT* gene (*DAT* mutant) (29). We used the DEseq2 method (33) to identify gene transcripts that were differentially expressed in either strain in response to AMPH treatment, as compared to exposure to vehicle alone (DE transcripts). We then compared the DE transcripts between the two strains. DEseq2 analysis showed profound transcriptional responses to AMPH in both strains (Figures 1A–D, statistically significant DE transcripts are shown in teal). We identified 717 DE transcripts corresponding to 362 unique genes in WT and 629 DE transcripts corresponding to 332 unique genes in DAT mutants (Figure 1, Supplementary Table 1), with approximately the same number of transcripts up and downregulated after the exposure (the MA plots in Figures 1A,B show similar number of transcripts above and below the zero line on the Y-axis).

As we were primarily interested in the DAT-dependent contribution to the transcriptional response to AMPH, we focused our further analysis on genes that no longer respond to AMPH in the absence of DAT, i.e., genes that are differentially expressed in the WT but not in the *DAT* mutant, when comparing the AMPH-treated groups to their respective vehicle controls. These are genes corresponding to the 308 transcripts that constitute the *w*^1118^ complement to the *w*^1118^/*DAT* intersection (shown in blue in Figures 1E,F) we refer to as DAT-dependent. We identified 409 transcripts that are differentially expressed in both strains (Figures 1E,F, red), 408 of which were changed in the same direction. Given that the *DAT* mutants do not express a functional DAT, we posit that the expression changes in these 408 shared transcripts (Figures 1E,F, red) are due to the effects of AMPH that are not mediated by DAT (DAT-independent), such as the responses to the actions of AMPH at other targets, including other neurotransmitter transporters (34). One gene, *takeout*, changed its expression in the opposite direction in the *DAT* mutant compared to control (Figure 1F, upper left quadrant). Lastly, we found 220 transcripts that only respond to AMPH in the *DAT* mutants and not in the controls (Figures 1E,F, green). These may reflect compensatory mechanisms that arise in response to the underlying hyperdopaminergic state in *DAT* mutants, such as the activation of other neurotransmitter systems that gain functional significance in the absence of DAT and the presence of AMPH.

### Functional Categories of Genes That Are Differentially Regulated by AMPH in a DAT-Dependent Manner

**Figure 2:**
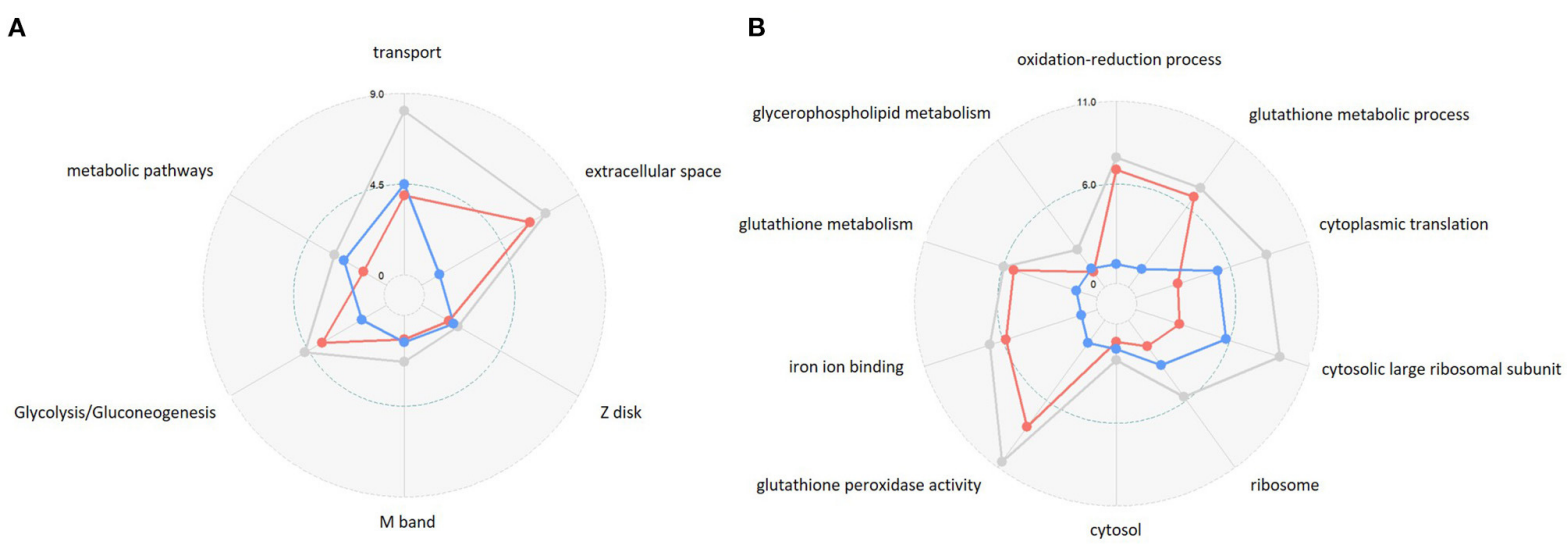
Comparison of the top GO terms that are enriched in DE genes in the WT strain. GO terms are compared by their *p*-values; the centers of the circles correspond to –log10(p) = 0, which increases outwards. **(A)** Downregulated genes; **(B)** upregulated genes. Gray: all DE genes in WT (these correspond to the 717 DE transcripts in Figure 1E, gray circle); red: DE genes that are shared between WT and *DAT* mutant (these correspond to the 409 transcripts depicted in red in Figures 1E,F); blue: DE genes that are unique to WT and not shared with *DAT* mutant (these correspond to the 308 transcripts depicted in blue in Figures 1E,F). The diagrams were created using the R package *ggradar* (36).

For functional interpretation of the DAT-dependent transcriptional effects of AMPH, we next compared known functions of the DAT-dependent genes to those of the DAT-independent genes, separately for upregulated and downregulated genes, to find significant differential functional enrichment [in KEGG pathways and Gene Ontology (GO) Biological Process (BP) categories (35)] (Supplementary Tables 2, 3). We found several functional terms that were differentially enriched when the group of DAT-dependent genes was compared with the DAT-independent group (Figure 2, Supplementary Figure 1). We also detected a number of common functional themes between these two gene sets, which could reflect the cumulative changes in gene activity in response to AMPH across different parts of the fly brain. Notably, even among the shared functional terms, we found significant differences in the distribution of the *p*-values between the two groups (Figure 2, compare blue to red). Taken together, the differential enrichment of the shared terms as well as the significant differences in overall functional themes suggest that the two gene lists, DAT-dependent and DAT–independent, were drawn from functionally distinct sets of genes representing the distinct actions of AMPH in the presence or absence of its primary molecular target.

The functional differences between DAT-dependent and DAT-independent genes were more pronounced among the upregulated genes (Figure 2, compare blue to red). Furthermore, upregulated genes had a higher number of specific differentially enriched functional annotation terms (as opposed to general ones) than downregulated genes, which did not show any particular predominant categories in the enrichment analysis (Figure 2A, Supplementary Figure 1E). Major functional categories that were predominantly enriched in the DAT-independent group, and therefore represented a common response to the drug between WT and *DAT* mutant, included oxidation-reduction process and glutathione metabolism (Figure 2, red), consistent with the presence of a general xenobiotic response to drug exposure (37).

**Figure 3:**
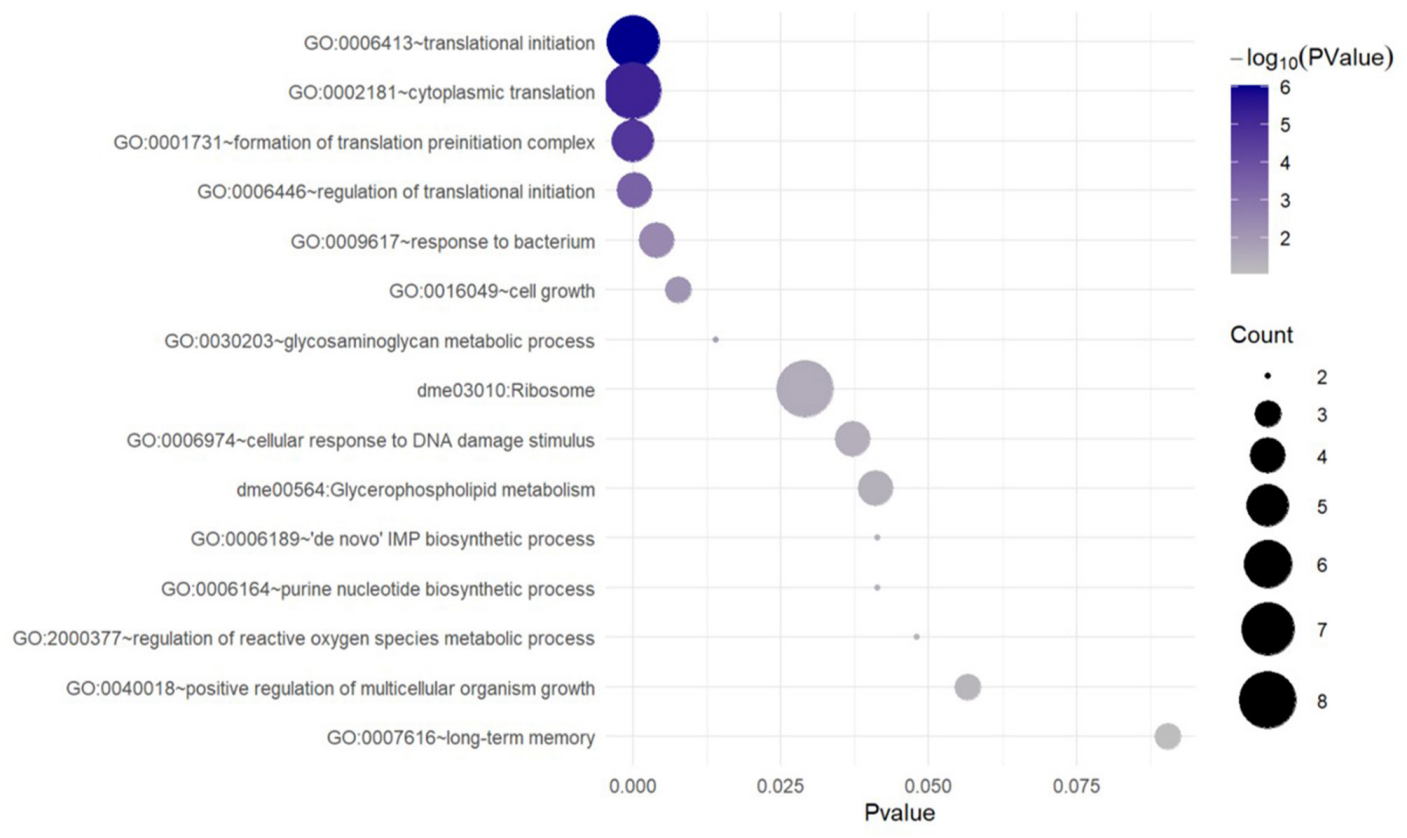
Unique GO and KEGG terms enriched with the genes that are upregulated in response to AMPH in a DAT-dependent manner. Functional category/term names are on the left. Circle sizes are proportional to the number of DE genes (gene counts), which were annotated as belonging to the corresponding functional category.

To characterize the functions specific to the DAT-dependent groups, we subtracted from the WT enrichment list the categories that were predominantly shared between WT and *DAT* mutant [categories that exhibited high significance (low *p*-values) in the DAT-independent set]. This resulted in a unique subset of terms specific to the genes that are upregulated in response to AMPH treatment in a DAT-dependent manner (Figure 3). This enrichment analysis showed that the predominant functional theme in the AMPH-induced DAT-dependent gene regulation was the activation of *de novo* mRNA translation. Among the upregulated genes were those encoding ribosomal subunits and components of the eukaryotic translation initiation factor 3 complex (eIF3) (Supplementary Table 3). We did not find any exclusively neuron-specific processes in this analysis, with the exception of genes encoding proteins that modulate cell-cell adhesion (in the downregulated group) and long-term memory (in the upregulated group); however, neither one of these general categories accumulated enough genes to provide more specific annotation that would allow for a more detailed functional classification (Supplementary Table 3).

### Convergent Data From a Genome-Wide Association Study (GWAS) of AMPH Sensitivity

**Figure 4:**
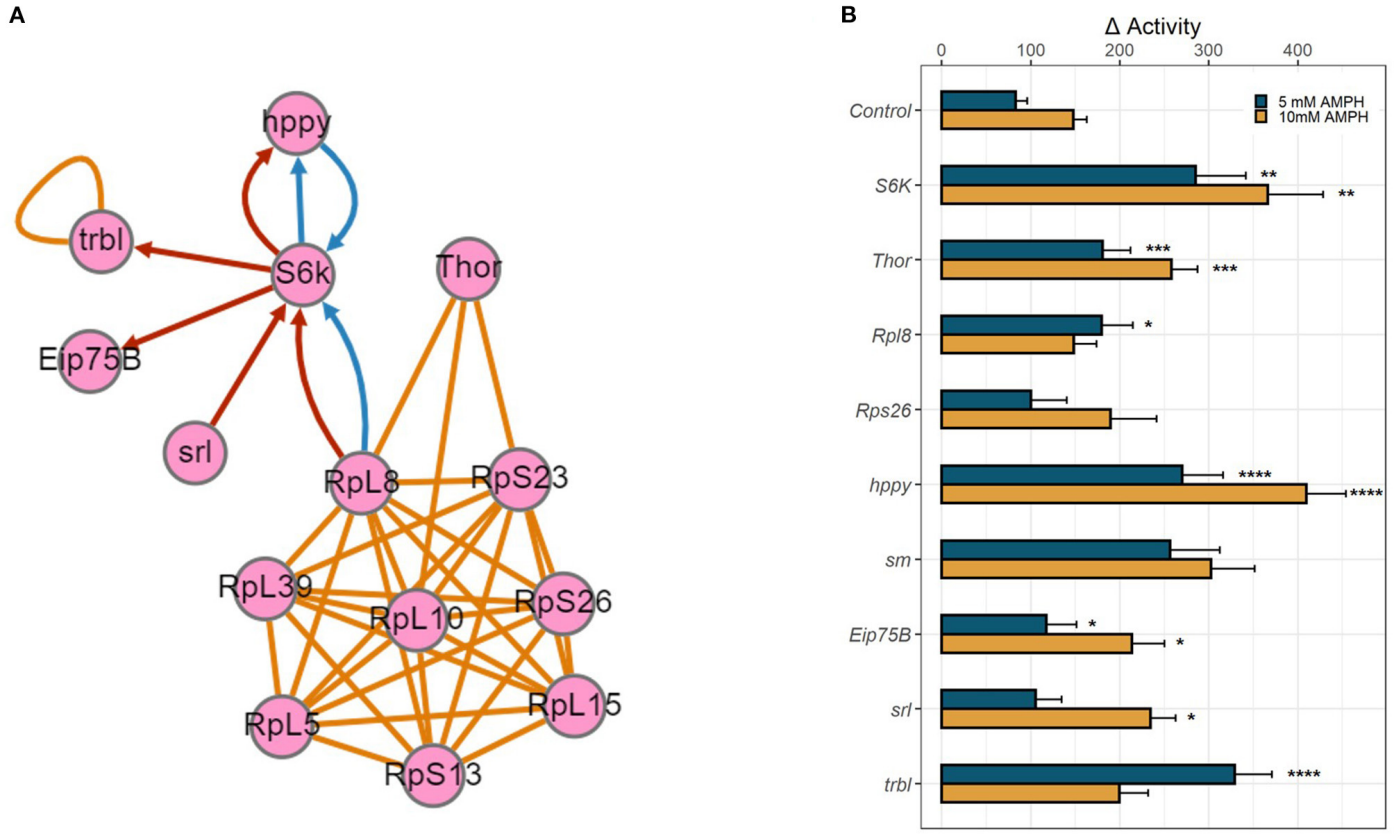
Functional validation of candidate genes. **(A)** Gene network depicting physical and genetic interactions between candidate genes identified in RNA-seq and GWA analyses. Orange lines indicate reported physical interaction, blue arrows indicate enhancing genetic interaction, and red arrows indicate suppressing genetic interaction. Network generated using the esyN webtool. *S6K* and *Thor* encode direct targets of the MTOR signaling pathway, S6 Kinase and 4E-BP, which are known to interact with ribosomal proteins to modulate mRNA translation. *hppy* encodes MAP4K3 which regulates the phosphorylation of S6K and 4E-BP. *Eip75B, srl*, and *trbl* all encode modulators of insulin-mTOR S6K signaling. **(B)** RNAi-mediated knockdown of candidate genes was targeted to dopamine neurons using TH-GAL4. Bar graphs depict change in activity in response to 5 mM AMPH (blue) or 10 mM AMPH (yellow). Error bars indicate SEM. Statistical significance was determined by Kruskal-Wallis ANOVA (*p* < 2e-16). Asterisks indicate pairwise significance compared to genotype control after AMPH treatment, as determined by *post-hoc* Dunn's Test with a Benjamini-Hochberg correction for multiple testing, ****p.adj < 0.0001, ***p.adj < 0.001, **p.adj < 0.01, *p.adj < 0.05.

The above transcriptomic analyses comparing AMPH-associated gene regulation in *DAT* mutants and their isogenic control (WT) were performed with the intention to prioritize for validation genes that were regulated only in the presence of DAT, as we anticipated those to be more relevant to the actions of AMPH at dopaminergic release sites. To further explore the functional relevance of the genes uncovered by this analysis, we mined data collected from an independent GWAS of AMPH-induced hyperactivity we conducted using the DGRP (Williams et al, *GWA uncovers a novel role for Ctr9 in AMPH sensitivity in* Drosophila, not yet published), a collection of inbred, fully sequenced fly lines (30). In addition to 3 SNPs that met the Bonferroni threshold of *p* < 10^−8^, our analyses found 288 SNPs within or near 123 genes associated with the response to AMPH at an empirical threshold of *p* < 10^−5^. Similarly to previous GWA studies performed with the DGRP (38), quantile-quantile plots of observed *p*-values against the distribution of expected *p*-values demonstrated significant deviation from linearity that supports the enrichment in true positive associations at or above this empirical threshold (Williams et al., *ibid*). Previous efforts by other groups using mutational analyses or targeted RNAi knockdown have validated 60–80% of gene associations that fall into this category (38). We posited that we could identify high-priority genes for further validation by looking for functional convergence between the genes we identified in our GWAS with those identified in the transcriptomic analyses presented above. Indeed, we found associated SNPs within or near several genes encoding ribosomal subunits (*RpL8, RpS13, RpS23*, and *RpS26*) (39) and *smooth* (*sm*), a heterogeneous nuclear ribonucleoprotein (hnRNP) with a role in mRNA splicing (40), which was also identified in the RNA-seq analysis. Pathway analysis further identified candidate genes from either study that encode proteins essential to the modulation of mRNA translation (Figure 4A). We found associated SNPs within or near the gene encoding the ribosomal protein S6 kinase (*S6k*), which promotes protein synthesis by phosphorylating the S6 ribosomal protein, and the gene *happyhour* (*hppy*), which encodes a member of the Ste20 family member (MAP4K3) that has been shown to be required for maximal phosphorylation of S6K and the eukaryotic translation initiation factor 4E binding protein (4E-BP1) (41). Notably, the fly has only one gene encoding a 4E binding protein, *Thor* (42, 43), which was also identified in our transcriptomic analyses above. Other candidate genes from the GWAS include *Eip75b*, the fly homolog of peroxisome proliferator-activated receptor γ (PPARγ), and *spargel* (*srl*), the homolog of PPARγ coactivator 1α (PGC-1α) (44, 45). PGC-1α has been previously shown to play a role in insulin-TOR signaling downstream of S6K (44). The RNA-seq analysis also identified *tribbles* (*trbl*), which encodes a Trib kinase previously shown to modulate Akt-mediated insulin signaling through S6K and PPARγ (46).

### Functional Validation of Candidate Genes

We employed targeted RNA interference (RNAi) using the GAL4/UAS system to knock down gene expression to test the role of the candidate genes identified in our analyses above. We targeted the expression of select RNAi constructs first pan-neuronally, using the elav-GAL4 driver (Supplementary Figure 2), and then in a more targeted manner using the dopamine neuron-specific TH-GAL4 driver (Figure 4B). Knockdown of several candidate genes, especially ribosomal proteins, was lethal when either GAL4 driver was utilized, precluding us from validating the role of these genes in the behavioral response to AMPH. For those that survived, knockdown of *Rpl8* significantly enhanced the response to 5 mM AMPH, whereas knockdown of *Rps26* had no effect. Knockdown of *S6K* led to a dramatic increase in AMPH-induced hyperactivity, using either GAL4 driver and at each AMPH concentration tested, suggesting that the gene plays a critical role in modulating the sensitivity to AMPH in general, and in dopamine neurons in particular (Figure 4B, Supplementary Figure 2). Similarly, knockdown of *Thor* (4E-BP), *hppy* (MAP43K), *Eip75B* (PPARγ), *srl* (PGC-1α), or *trbl* significantly enhanced the response to AMPH (Figure 4B, Supplementary Figure 2). These findings suggest a specific role for the S6K signaling pathway in dopamine neurons in modulating the initial sensitivity to AMPH and also point to a potential role for the insulin signaling pathway in regulating this process.

## Discussion

Understanding the genetic and molecular mechanisms underlying behavioral disorders, such as substance use and abuse, is critical for developing targeted therapeutic strategies to treat these disorders. Next-generation sequencing has greatly facilitated transcriptomic and genomic analyses, thereby allowing for unbiased approaches to identifying novel genes and gene pathways that mediate the actions of drugs of abuse. However, the direct functional implication of candidate genes remains challenging, especially in rodent models where *in vivo* gene validation can be costly and laborious. To effectively prioritize genes that modulate AMPH action, we analyzed the convergence of a combination of behavioral, transcriptomic, and genomic datasets, and followed up by high-throughput targeted validation in *Drosophila*.

Given that DAT is the principal molecular target of AMPH (4, 5), we first analyzed the transcriptional response to the psychostimulant in fly brains in the presence or absence of DAT. We focused on genes that are differentially regulated in response to the psychostimulant in a DAT-dependent manner, as we hypothesize that they are mechanistically linked to the behavioral response to AMPH, which is also DAT-dependent. Using functional enrichment analysis, we found that the major affected process was the upregulation of genes that govern *de novo* mRNA translation. We then examined whether genes that display similar ontology are enriched in an independent GWAS dataset. This approach allowed us to prioritize GWAS hits that met the empirical threshold of *p* < 10^−5^ but fell short of meeting the more stringent Bonferroni significance. Consistent with the results of the RNA-seq analysis, we identified several genes encoding ribosomal proteins, in addition to several modulators of mRNA translation. By exploring the functional convergence between transcriptomic and genomic data, we were able to identify genes that confer AMPH sensitivity via mechanisms downstream of the transcriptional response to the drug. The existence of such convergence suggests that the underlying genetic architecture can have a significant impact on signaling pathways triggered by drug exposure. Other functional themes identified by our GWAS include neurodevelopment, cell adhesion, and control of locomotion and sleep, among others (Williams et al., *ibid*).

Taking advantage of the high-throughput gene targeting tools available in *Drosophila*, we validated the role of several of these genes, including the fly homologs of *S6K* and *4E-BP*, which are direct targets of the mammalian Target of Rapamycin in Complex 1 (mTORC1). Remarkably, even though we measured transcripts from whole heads, we were able to identify genes that regulate AMPH sensitivity specifically in dopamine neurons, which represent a tiny fraction of neurons in the central nervous system. We believe that this success in identifying genes that are critical to the dopaminergic response to AMPH was facilitated by our prioritizing DAT-dependent transcriptional changes. The classification of DAT-dependent vs. DAT-independent transcriptional changes is imperfect, given the compensatory changes associated with the mutant and time course of drug action. This is in part supported by our findings that pan-neuronal knockdown of some of the candidate genes leads to an even more enhanced AMPH response compared to dopamine neuron-specific knockdown, suggesting a role for these genes in neurons pre- and/or postsynaptic to dopamine neurons, or in other neuronal circuits altogether. Nonetheless, we believe that this initial prioritization helped focus our subsequent functional analyses and validation to identify gene pathways in dopamine neurons that modulate the response to AMPH's actions at DAT. Future studies will be needed to explore the role of these pathways in other neuronal populations.

In recent years, a wealth of data has implicated the kinase mTORC1 (mammalian/mechanistic Target of Rapamycin in Complex 1) as an essential mediator of protein synthesis (47), including dendritic translation of synaptic proteins (48, 49). In this role, mTORC1 is known to promote neuroadaptations in response to key signaling events, such as those that are induced by drugs of abuse (12, 13, 50). mTORC1 targets S6K and 4E-BP (51), candidate genes we identified that are critical for the initiation and elongation of mRNA translation. Our data further showed that targeted RNAi knockdown of either protein in dopamine neurons dramatically enhanced the response to AMPH. More work needs to be done to explicate the mechanisms underlying the roles of these genes in AMPH sensitivity. It would be particularly interesting to perform cell-specific ribosome profiling (52) in order to delineate the translational network activated in response to AMPH in dopamine neurons, as well as other neuronal subtypes, to begin to understand the link between the observed transcriptional changes and behavioral phenotype.

Previous studies have implicated mTOR signaling in the actions of psychostimulants, but these mostly focused on pharmacological inhibition or genetic knockdown or deletion of *mTORC1* and downstream effectors in adulthood, which attenuated psychostimulant-induced reward and reinforcement behavior (53–58). Notably, our knockdown approach targets candidate genes early during development. Thus, one possible explanation for the different results is that S6K signaling plays a role in the neurodevelopment of dopamine neurons in ways that influences the response to AMPH later in life, and that this role may be distinct from its function in the acute response to AMPH in adulthood. Consistent with this hypothesis, our data also showed that dopamine neuron-specific knockdown of *srl* (dPGC-1) (44) and its coactivator *Eip75b* (dPPARγ) (45), transcriptional regulators that act downstream of the insulin/Akt/TOR pathway (44), enhanced the response to AMPH. PGC-1 and PPARγ have been studied as therapeutic targets in Parkinson's disease (59, 60) and have been shown to confer neuroprotective effects in dopamine neurons (61). Interestingly, the Trib kinase encoded by the candidate gene *trbl* has been shown to modulate Akt-mediated insulin signaling through S6K and PPARγ (46). In light of a series of studies implicating insulin as a regulator of dopamine uptake and release (62–64), our data suggest a working model in which insulin signaling in dopamine neurons acts through S6K during neurodevelopment to modulate AMPH sensitivity, possibly by altering the functional expression of DAT, its dopamine reuptake capacity, or its ability to efflux dopamine in response to AMPH. This is also consistent with a study showing that insulin promotes dopamine neuron differentiation through PI3K/Akt/mTOR-dependent S6K signaling in human neural stem cells (65). Further studies will be needed to test this hypothesis, using tools readily available in flies for temporal control of gene knockdown (66), which would enable comparison of the effect of knockdown during development to knockdown in adulthood. It will also be interesting to explore whether the genes identified effect changes in AMPH sensitivity by modulating autophagy, another major cellular process controlled by mTOR (50). Previous studies have shown that inhibition of mTOR induces formation of autophagic vacuoles in presynaptic dopamine terminals, leading to decreased size of axonal profiles, synaptic vesicle numbers, and evoked dopamine release (67). Several studies have also shown that autophagy mediates psychostimulant-induced neurotoxicity (68–70). More recently, a study showed that low, non-toxic levels of cocaine also induce neuronal autophagy *in vitro* and *in vivo*, and that inhibitors of autophagy blunt conditioned place preference in mice (71). Interestingly, cocaine-induced autophagy was also shown to induce DAT degradation in the nucleus accumbens of mice, and it would be interesting to determine if mTOR is linked mechanistically to this process as well (71).

Taken together, our data demonstrate the power of *Drosophila* as a genetic model that facilitates high-throughput behavioral screens, combined with GWAS and whole transcriptome sequencing, to identify, prioritize, and validate candidate genes that can be subsequently evaluated in rodent models of self-administration.

## Materials and Methods

### Fly Stocks and Transgenic *Drosophila* Lines

All fly strains were reared on a standard corn meal, yeast, molasses, and agar medium at 25°C and 45–47% humidity under a 12:12 h light:dark cycle. An isogenic *w*^1118^ fly strain (Exelixis strain A5001, BL-6326) was used as the control. The *DAT* mutants (*dDAT*^*fmn*^) were a gift from Dr. K. Kume (Kumamoto, Japan) (29) and were back-crossed to the *w*^1118^ isogenic strain for 7 generations. These mutants have the 5′ portion of a roo transposon inserted into intron 6 of the *dDAT* gene resulting in an in-frame stop codon (29). All RNAi lines were driven by the TH-GAL4 driver (72), a gift from Dr. S. Birman (Paris, France). Transgenic RNAi strains were obtained from Bloomington Stock Center (Stocks: GFP RNAi #9330, Rpl8 RNAi #50610, Rps26 RNAi #33393, S6K RNAi #42572, Thor RNAi #80427, trbl RNAi #60007, hppy RNAi #53884, srl RNAi #33915, Eip75B RNAi #35780, Sm RNAi #64524).

### Behavioral Assay

Flies were aged for 7 days after eclosion, housed in vials containing standard medium, and entrained to a 12:12 h light:dark regime under rearing conditions. Individual aged male flies were then anesthetized briefly with CO_2_ and placed in polycarbonate tubes containing food consisting of 1% agar and 3% sucrose delivered in water (vehicle) or AMPH solution (10 mM) (Sigma, A5880). Flies were continuously monitored for movement by four infrared beams evenly distributed across the tube using Trikinetics *Drosophila* Activity Monitors (TriKinetics, Waltham, MA). Locomotor activity was measured by recording infrared beam crossings (activity counts) by individual flies totaled in 2 min bins. All experiments were carried out in a designated behavior room under LD conditions at 25°C and ~45–50% humidity with *ad libitum* access to food (Vehicle or AMPH). Animals that died within the first 12 h of the experiment were excluded from the analysis.

Output files were analyzed in R using the previously published Rethomics framework (73). The mean activity was calculated by binning activity counts over 60 min and averaging across the first night of recording (0–12 h) for each individual animal.

Bar graphs were generated using ggplot2 (36, 74) and represent the change in response to AMPH for each group. Error bars indicate SEM. When the assumption of homogeneity of variance for the data was not met, statistical significance was determined by Kruskal-Wallis ANOVA. Asterisks indicate pairwise significance compared to genotype control after AMPH treatment, as determined by *post-hoc* Dunn's Test with a Benjamini-Hochberg correction for multiple testing, ^****^p.adj < 0.0001, ^***^p.adj < 0.001, ^**^p.adj < 0.01, ^*^p.adj < 0.05.

### RNA-Seq

PolyA+ selected libraries were produced from individual whole *Drosophila* brains (4 heads per group). Specifically, total RNA was prepared using the RNAqueous-Micro kit (Ambion) followed by polyA+ mRNA isolation with the Dynabeads mRNA DIRECT Micro kit (Ambion) according to the manufacturer's protocols. Barcoded libraries (Ion XpressRNA) were made using the Ion Total RNA-Seq Kit v2 (Fisher Scientific), PCR-amplified, and quantified with Agilent Bioanalyzer (Agilent). The resulting libraries were pooled and sequenced on the Ion OneTouch 2 System using P1.1.17 chips and Ion HiQ chemistry. Data was collected with Ion Suite (version 4.4.3. 1). Reads were mapped to UCSC dm3 reference genome with tmap-f3 in Torrent Suite (v4.4.3). The raw and processed RNA-Seq data were submitted to the NCBI Gene Expression Omnibus with the Accession Number GSE196162.

### Differential Gene Expression Analysis

Differential gene expression analysis based on the negative binomial distribution model was performed using the DESeq2 R package (33). In this analysis, we first found the differential expression in each AMPH treatment group (namely, the isogenic *w*^1118^ flies (WT) and *DAT* mutant flies) against their respective untreated controls. The differentially expressed genes in each comparison were selected at the adjusted *p*-value (Benjamini and Hochberg) of <0.1, followed by the analysis of similarities between the resulting gene lists.

### Gene Ontology (GO) and Functional Enrichment Analysis

Functional classification of candidate genes was performed using established GO terms and canonical pathways available in the Database for Annotation, Visualization and Integrated Discovery (DAVID) (35). Enrichment in functional GO categories and pathways (from the annotated pathway collections and tools such as KEGG and GO BP terms was assessed using Fisher's exact and hypergeometric tests available through DAVID. Genetic and physical interactions between candidate genes were identified and graphed using the esyN webtool (which uses the Flybase Gene Report and Flybase Interaction Report to identify functional gene networks) (75), and by mining the literature for functional interaction between mammalian homologs of candidate genes.

## Data Availability Statement

The original contributions presented in the study are publicly available. This data can be found here: https://www.ncbi.nlm.nih.gov/, accession: GSE196162.

## Author Contributions

CK, SK, CH, DG, and JJ designed the experiments. SK designed data analysis tools for functional enrichment analysis. CK, BW, RP, and YZ performed the experiments. CK, BW, IM, QY, SK, and CH contributed analysis tools and performed data analysis. CK, BW, IM, SK, and JJ discussed the results and interpretation of the data and wrote the manuscript. All authors contributed to the article and approved the submitted version.

## Funding

This work was supported by U01 DA042233 (CK, BW, YZ, and JJ), a Seed Grant from the Columbia University Data Science Institute (SK and IM), and the Intramural Research Program of the National Institute on Alcohol Abuse and Alcoholism (RP, QY, CH, and DG).

## Conflict of Interest

The authors declare that the research was conducted in the absence of any commercial or financial relationships that could be construed as a potential conflict of interest.

## Publisher's Note

All claims expressed in this article are solely those of the authors and do not necessarily represent those of their affiliated organizations, or those of the publisher, the editors and the reviewers. Any product that may be evaluated in this article, or claim that may be made by its manufacturer, is not guaranteed or endorsed by the publisher.

## Supplementary Material

Supplementary Figure 1 Prevalent Biological Process categories (GO BP terms) and KEGG pathways (dme term) enriched in genes differentially expressed in WT AMPH response. **(A)** Downregulated genes; **(B)** upregulated genes. All enriched functional annotation terms were selected at the *p*-value threshold below 0.05 and sorted in increasing order from left to right in the diagrams; **(C)** terms enriched with downregulated genes, which are also downregulated in the *DAT* mutant; **(D)** terms enriched with upregulated genes, which are also upregulated in the *DAT* mutant strain. These genes are red in Figures 2A,B. As before, all enriched functional annotation terms were selected at the *p*-value threshold below 0.05 and sorted in increasing order from left to right in the diagrams; **(E)** terms enriched with downregulated genes, which are not differentially expressed in the *DAT* mutant; **(F)** Terms enriched with upregulated genes, which are not differentially expressed in the *DAT* mutant. These genes are blue in Figures 2A,B. As before, all enriched functional annotation terms were selected at the *p*-value threshold below 0.05 and sorted in increasing order from left to right in the diagrams.

Supplementary Figure 2 RNAi-mediated knockdown of candidate genes was targeted to all neurons using elav-GAL4. Bar graphs depict change in activity in response to 5 mM AMPH (blue) or 10 mM AMPH (yellow). Error bars indicate SEM. Statistical significance was determined by Kruskal-Wallis ANOVA (*p* < 2e-16). Asterisks indicate pairwise significance compared to genotype control after AMPH treatment, as determined by *post-hoc* Dunn's Test with a Benjamini-Hochberg correction for multiple testing, ^****^*p*.adj < 0.0001, ^***^*p*.adj < 0.001, ^**^*p*.adj < 0.01, ^*^*p*.adj < 0.05.

Supplementary Table 1 List of transcripts differentially expressed in response to AMPH in WT and in *DAT* mutant.

Supplementary Table 2 GO terms and functional enrichment analysis for DAT-independent genes. **DATindependent** is the list of DE genes common between WT and *DAT* mutant; **up** and **dn** list the FBtr recognized by DAVID for up- and downregulated genes; **upANNO** and **dnANNO** contain all significant annotations (*p* < 0.1) in all functional databases used in DAVID.

Supplementary Table 3 GO terms and functional enrichment analysis for DAT-dependent genes. **DATdependent** is the list of DE genes unique to WT; **up** and **dn** list the FBtr recognized by DAVID for up- and downregulated genes; **upANNO** and **dnANNO** contain all significant annotations (*p* < 0.1) in all functional databases used in DAVID. Several functionally related categories share common genes, e.g., GO: 0006413~translational initiation; GO: 0001731~formation of translation preinitiation complex; GO: 0005852~eukaryotic translation initiation factor 3 complex. There are no shared genes for unrelated functional categories.
