# Supplementary figures and images for: Functional Genomic Analysis of Amphetamine Sensitivity in Drosophila
Source: Front Psychiatry. 2022 Feb 18;13:831597. doi: 10.3389/fpsyt.2022.831597 (PMC8894854; doi:10.3389/fpsyt.2022.831597)

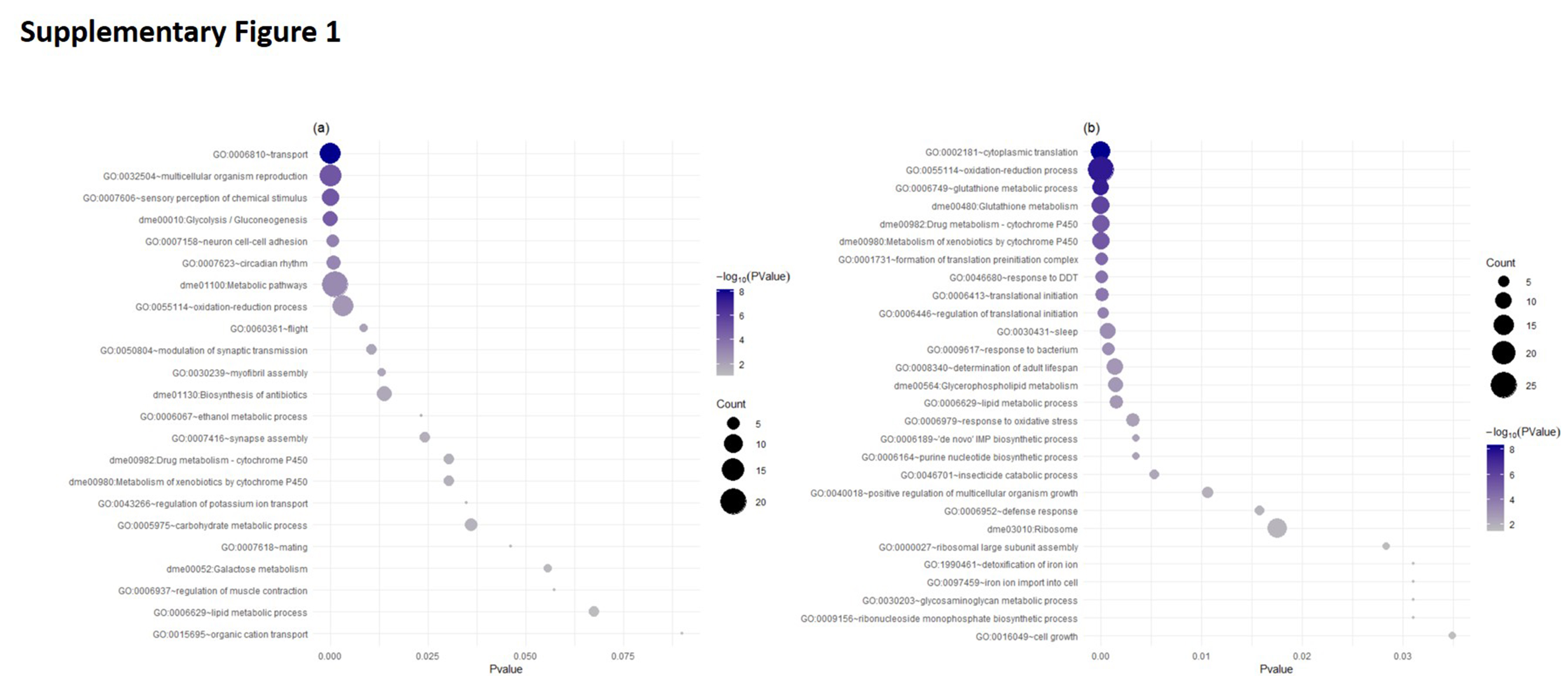

Supplement: Supplementary Figure 1 — Prevalent Biological Process categories (GO BP terms) and KEGG pathways (dme term) enriched in genes differentially expressed in WT AMPH response. (A) Downregulated genes; (B) upregulated genes. All enriched functional annotation terms were selected at the p-value threshold below 0.05 and sorted in increasing order from left to right in the diagrams; (C) terms enriched with downregulated genes, which are also downregulated in the DAT mutant; (D) terms enriched with upregulated genes, which are also upregulated in the DAT mutant strain. These genes are red in Figures 2A,B. As before, all enriched functional annotation terms were selected at the p-value threshold below 0.05 and sorted in increasing order from left to right in the diagrams; (E) terms enriched with downregulated genes, which are not differentially expressed in the DAT mutant; (F) Terms enriched with upregulated genes, which are not differentially expressed in the DAT mutant. These genes are blue in Figures 2A,B. As before, all enriched functional annotation terms were selected at the p-value threshold below 0.05 and sorted in increasing order from left to right in the diagrams. [file Image_1.JPEG]

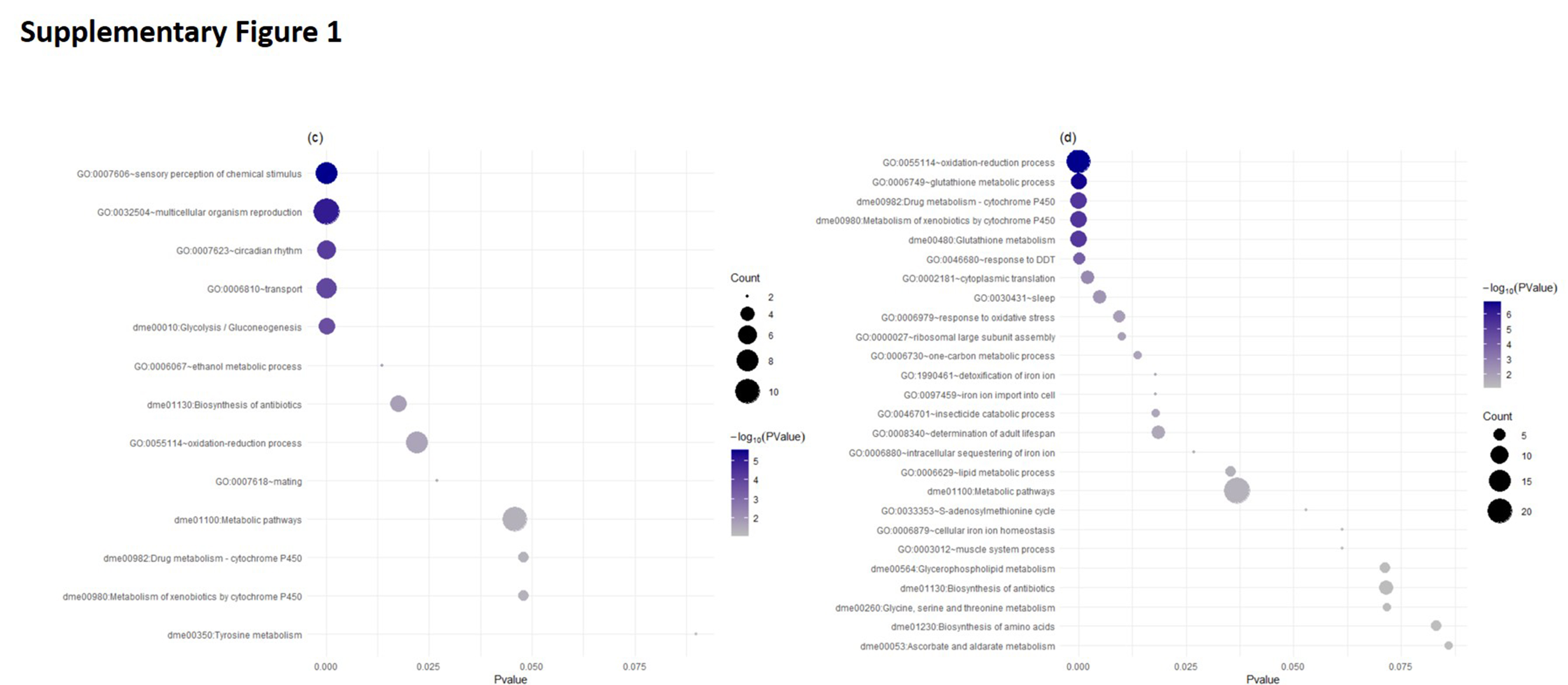

Supplement: Supplementary Figure 2 — RNAi-mediated knockdown of candidate genes was targeted to all neurons using elav-GAL4. Bar graphs depict change in activity in response to 5 mM AMPH (blue) or 10 mM AMPH (yellow). Error bars indicate SEM. Statistical significance was determined by Kruskal-Wallis ANOVA (p < 2e-16). Asterisks indicate pairwise significance compared to genotype control after AMPH treatment, as determined by post-hoc Dunn's Test with a Benjamini-Hochberg correction for multiple testing, ****p.adj < 0.0001, ***p.adj < 0.001, **p.adj < 0.01, *p.adj < 0.05. [file Image_2.JPEG]

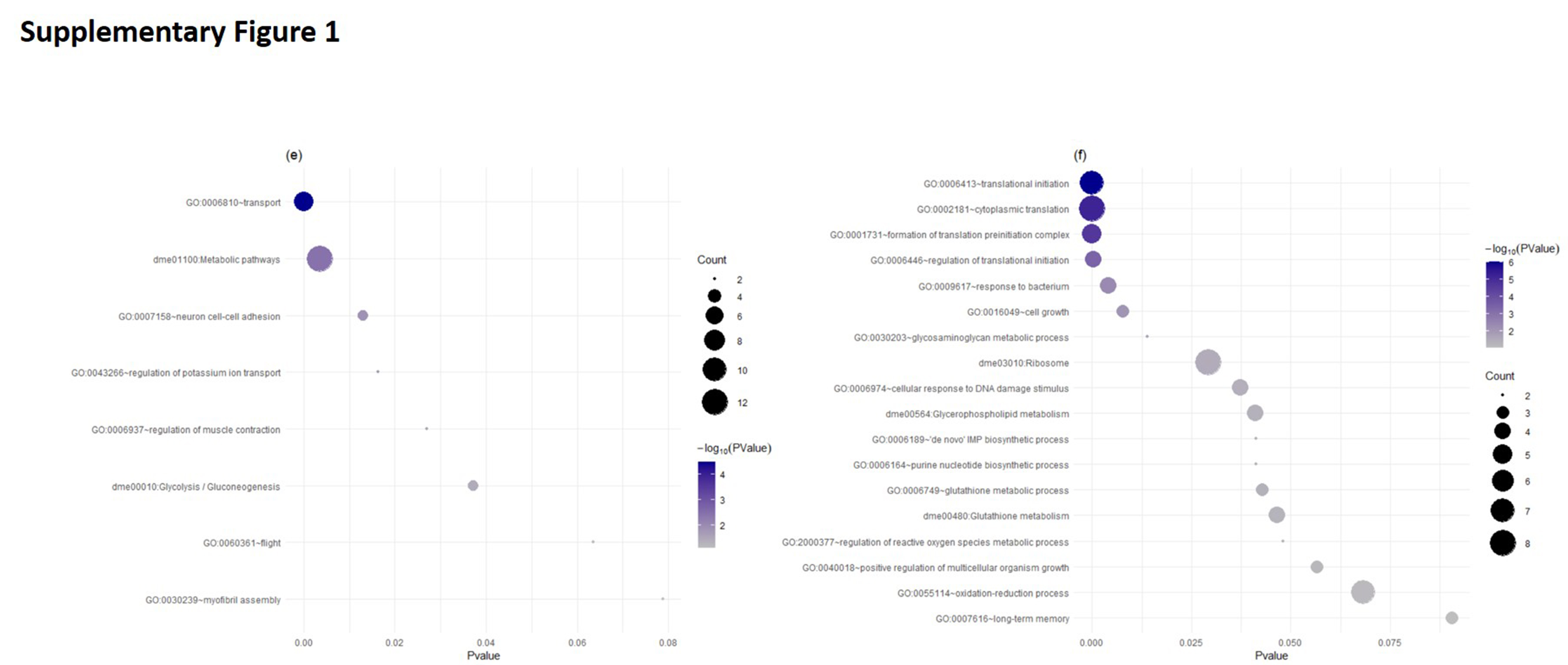

Supplement: Supplementary file 6 [file Image_3.JPEG]

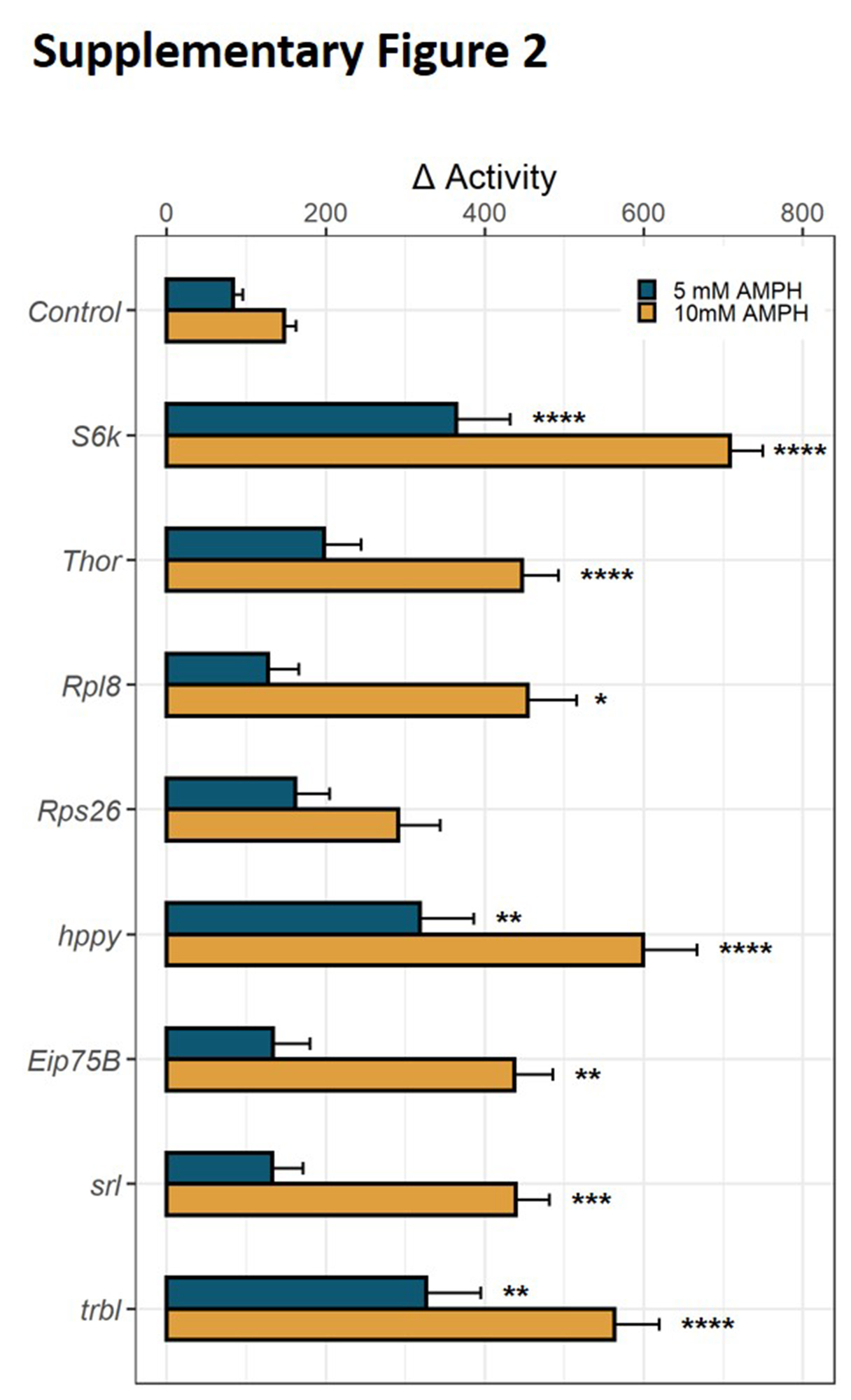

Supplement: Supplementary file 7 [file Image_4.JPEG]
